# Progress and Inspiration of Social Capital in the Prevention and Control of Diabetes

**Published:** 2020-01

**Authors:** Hengjun JIANG, Xingling CHEN, Yuxiu HU, Feng TAO

**Affiliations:** 1 Scientific Research Department, The First Affiliated Hospital, College of Medicine, Zhejiang University, Hangzhou, 310003, Zhejiang, China; 2 The Second Affiliated Hospital of Zhejiang Chinese Medical University, Hangzhou, 310005, Zhejiang, China

## Dear Editor-in-Chief

Social capital is one of the important factors affecting population health (1). As an intangible social resource, it has important practical significance in the prevention and treatment of diabetes. Using the system review method, we have reviewed previous published literature and analyzed the concept of social capital and its application to diabetes prevention and treatment. This study aimed to provide information and enlightenment to effectively mobilize all resources that promote diabetes prevention and treatment. The study was significant and meaningful in the relationship between social capital and diabetes, and it is the first study to conduct a review of this relationship.

We retrieved 2329 articles and 2029 records were included after removing duplicates. Of these, 98 records were included based on the titles and abstracts. Overall, 83 articles were excluded because of full texts and finally, 15 articles containing 15 studies were included.

In addition, diabetes has been shown to be closely related to social and economic status (2,3). In recent years, the social determinant of health theory has gained more attention, and scholars have expanded the study vision to the field of social capital for the purpose of development and utilization. It obviously has important practical significance and a positive role for the effective use of existing resources for the prevention and control of chronic diseases, and which promotes greater social efficacy.

Even though diabetes at present cannot be completely cured, it can be controlled with the active participation of patients using effective self-management. The exploratory individual-level study identified a potentially strong relationship between social capital and diabetes self-management in Chinese adults (4).

A total diabetes prevention strategy involves the community in health promotion. Social capital at the macro level is closely linked to collective action and public policy (5,6). By producing a high degree of reciprocal trust, norms and social justice, it improves recognition of the general population and the participation of the social network to encourage collectivity and consistency in their behaviors.

Furthermore, living in a harmonious and united community provides more access to healthy food, safe sports areas and high-quality medical services, which are especially important for the prevention of diabetes. When the feeling of mutual assistance rises to a spiritual and moral code standard, it will greatly enhance the degree of social participation. In an information society, we should make full use of mass media including internet, television, radio and newspapers, to encourage the knowledge of diabetes prevention and to maximize the use all social resources for improving the prevention and control of diabetes.

As an intangible social resource, social capital has important practical significance in diabetes patients and can provide inspiration and enlightenment for the prevention and control of diabetes.

## References

[1] Menke A Casagrande S Geiss LM Cowie CC (2015). Prevalence of and trends in diabetes among adults in the United States, 1988– 2012. JAMA, 314(10):1021–9. 26348752 10.1001/jama.2015.10029

[2] Hu F Niu L Chen R (2015). The association between social capital and quality of life among type 2 diabetes patients in Anhui province, China: a cross-sectional study. BMC Public Health 15:786. 26276271 10.1186/s12889-015-2138-y PMC4542125

[3] Chen Ren Tao Feng Ying Ma (2014). Associations between Social Support and Condom Use among Commercial Sex Workers in China: A Cross-sectional Study. PLoS One 9(12):e113794. 25436910 10.1371/journal.pone.0113794 PMC4249969

[4] Riumallo-Her CJ Kawachi I Avendano M (2014). Social capital, mental health and biomarkers in Chile: Assessing the effects of social capital in a middle-income country. Soc Sci Med, 105:47–58. 24495808 10.1016/j.socscimed.2013.12.018 PMC4089037

[5] Joensen LE Filges T Willaing I (2016). Patient perspectives on peer support for adults with type 1 diabetes: a need for diabetes-specifc social capital. Patient Prefer Adherence, 10:1443–51. 27536076 10.2147/PPA.S111696 PMC4977079

[6] Tao Feng (2015). Research on the Associations of social capital and self-managing behaviors among diabetes patients. Hefei: Anhui Medical University; http://cdmd.cnki.com.cn/Article/CDMD-10366-1015329189.htm

